# S86. EXAMINING REASONING BIASES IN SCHIZOPHRENIA USING A MODIFIED “JUMPING TO CONCLUSIONS” TASK

**DOI:** 10.1093/schbul/sby018.873

**Published:** 2018-04-01

**Authors:** Hans Klein, Amy Pinkham

**Affiliations:** University of Texas at Dallas

## Abstract

**Background:**

The Jumping To Conclusion (JTC) bias has been extensively studied in relation to schizophrenia and persecutory delusions. It is suggested that performance on the traditional JTC task relates to a pervasive bias to make decisions quickly, contributing to delusion formation. However, the mechanisms underlying performance on this task, as well as the relationship between the JTC bias and other reasoning biases implicated in delusional ideation, is not fully understood. We examined the relationship between several biases believed to be involved in delusion formation and maintenance to further clarify potential co-occurrences of these biases and their relation to delusional ideation.

**Methods:**

In order to assess the co-occurrence of reasoning biases in decision making, we modified the traditional JTC task in order to assess a number of previously identified biases that may be implicated in delusion formation and maintenance. 46 participants with schizophrenia and 46 healthy controls completed two versions of the modified task utilizing neutral (blue and red beads in a jar) and salient (negative and positive comments in a list) stimuli, both with 60:40 ratios.

**Results:**

2 x 2 mixed ANOVAs were performed on each of the modified variables using group [patients vs. controls] as a between subjects variable and task type [neutral vs. salient] as a within subject variable. We replicated previous findings of main effects of a JTC bias for group, F(1, 90) = 4.149, p = .045, η2p = .044, and task type, F(1, 90) = 4.724, p = .032, η2p = .050 such that patients showed a greater JTC bias, and in both groups, the JTC bias was more pronounced for the salient task. However, a main effect of group was also evident for number of illogical judgments, F(1, 90) = 11.596, p = .001, η2p = .114, indicating that patients showed greater difficulty in probabilistic reasoning. When controlling for probabilistic reasoning ability, the group main effect for the JTC bias disappeared, F(1,89) =0.169, p = 0.682, η2p = 0.002. None of our modified variables significantly correlated with symptom severity within our patient population.

**Discussion:**

While we were not able to correlate our modified variables with symptoms of schizophrenia, we were able to observe a pattern of group differences that may help further understand decision-making processes in individuals with schizophrenia. Our findings that faulty probability assessment accounts for the JTC bias indicates that the traditional JTC bias task may not represent an inherent hasty decision making bias, but rather an inability to fully understand and execute the stated goals of the task. These results call into question the current understanding of the JTC bias and the independence of this bias apart from the cognitive demands of the task.

